# Carbohydrate quality, fecal microbiota and cardiometabolic health in older adults: a cohort study

**DOI:** 10.1080/19490976.2023.2246185

**Published:** 2023-08-23

**Authors:** Alessandro Atzeni, Stephanie K. Nishi, Nancy Babio, Clara Belzer, Prokopis Konstanti, Jesús Vioque, Dolores Corella, Olga Castañer, Josep Vidal, Isabel Moreno-Indias, Laura Torres-Collado, Eva M. Asensio, Montserrat Fitó, Ana Maria Gomez-Perez, Alejandro Arias, Miguel Ruiz-Canela, Frank B. Hu, Francisco J. Tinahones, Jordi Salas-Salvadó

**Affiliations:** a Departament de Bioquímica i Biotecnologia, Universitat Rovira i Virgili, Unitat de Nutrició Humana, Reus, Spain; b Human Nutrition Unit, Institut d’Investigació Sanitària Pere Virgili (IISPV), Reus, Spain; c CIBER de Fisiopatología de la Obesidad y Nutrición, Instituto de Salud Carlos III, Madrid, Spain; d Toronto 3D (Diet Digestive Tract and Disease) Knowledge Synthesis and Clinical Trials Unit, Toronto, Canada; e Clinical Nutrition and Risk Factor Modification Centre, St. Michael’s Hospital, Unity Health Toronto, Toronto, Canada; f Laboratory of Microbiology, Wageningen University, Wageningen, The Netherlands; g CIBER de Epidemiología y Salud Pública (CIBERESP), Instituto de Salud Carlos III, Madrid, Spain; h Instituto de Investigación Sanitaria y Biomédica de Alicante, Universidad Miguel Hernández (ISABIAL-UMH), Alicante, Spain; i Department of Preventive Medicine, University of Valencia, Valencia, Spain; j Unit of Cardiovascular Risk and Nutrition, Institut Hospital del Mar de Investigaciones Médicas Municipal d’Investigació Médica (IMIM), Barcelona, Spain; k CIBER Diabetes y Enfermedades Metabólicas (CIBERDEM), Instituto de Salud Carlos III (ISCIII), Madrid, Spain; l Department of Endocrinology, Institut d’Investigacions Biomédiques August Pi Sunyer (IDIBAPS), Hospital Clinic, University of Barcelona, Barcelona, Spain; m Department of Endocrinology and Nutrition, Virgen de la Victoria University Hospital, the Biomedical Research Institute of Malaga and Platform in Nanomedicine (IBIMA-BIONAND Platform), University of Malaga, Malaga, Spain; n Department of Psychiatry, Donders Institute for Brain, Cognition and Behaviour, Radboud University Medical Center, Nijmegen, The Netherlands; o Department of Human Genetics, Donders Institute for Brain, Cognition and Behaviour, Radboud University Medical Center, Nijmegen, The Netherlands; p Department of Preventive Medicine and Public Health, University of Navarra, Pamplona, Spain; q IdiSNA, Navarra Institute for Health Research, Pamplona, Spain; r Department of Nutrition, Harvard T. H. Chan School of Public Health, Boston, MA, USA; s Channing Division for Network Medicine, Department of Medicine, Brigham and Women’s Hospital and Harvard Medical School, Boston, MA, USA

**Keywords:** Carbohydrate quality, fecal microbiota, cardiovascular disease, Mediterranean diet

## Abstract

The impact of carbohydrate quality, measured by the carbohydrate quality index (CQI), on gut microbiota and health has been scarcely investigated. The aim of this study was to cross-sectionally and longitudinally explore the relationships between CQI, fecal microbiota, and cardiometabolic risk factors in an elderly Mediterranean population at high cardiovascular risk. At baseline and 1-year, CQI was assessed from food frequency questionnaires data, cardiometabolic risk factors were measured, and fecal microbiota profiled from 16S sequencing. Multivariable-adjusted linear regression models were fitted to assess the associations between tertiles of baseline CQI, fecal microbiota, and cardiometabolic risk factors at baseline, and between tertiles of 1-year change in CQI, 1-year change in fecal microbiota and cardiometabolic risk factors. Cross-sectionally, higher CQI was positively associated with Shannon alpha diversity index, and abundance of genera *Faecalibacterium* and *Christensenellaceae* R7 group, and negatively associated with the abundance of *Odoribacter*, and uncultured *Rhodospirillales* genera. Some of these genera were associated with higher glycated hemoglobin and lower body mass index. In addition, we observed a positive association between CQI, and some pathways related with the metabolism of butyrate precursors and plants-origin molecules. Longitudinally, 1-year improvement in CQI was associated with a concurrent increase in the abundance of genera *Butyrivibrio*. Increased abundance of this genera was associated with 1-year improvement in insulin status. These observations suggest that a better quality of carbohydrate intake is associated with improved metabolic health, and this improvement could be modulated by greater alpha diversity and abundance of specific genera linked to beneficial metabolic outcomes.

## Introduction

Carbohydrates are a relevant component of the diet, providing an important source of energy for the body.^1^ They have complex chemical structures that constitute a rich physiological function of the living system. Globally, there are recommended daily amounts for carbohydrate intake, generally ranging from 45 to 65% of total energy^2^ and worldwide intakes generally align with these recommendations.^1^ While historically the amount of carbohydrate consumption has been the focus of research and guidelines, not all carbohydrates are equal in terms of their impact on human health. Therefore, carbohydrate quality has been emerging over the past decades as an important factor to consider in dietary recommendations.^3–6^ As carbohydrate quality is multidimensional, a carbohydrate quality index (CQI) has been defined integrating four parameters into a single score including the ratio of solid carbohydrates to total carbohydrates, dietary fiber, glycemic index, and the ratio of whole grains to total grains.^7^ This CQI has been suggested as a useful tool in evaluating the micronutrient adequacy of the diet.^3,7^ Further, a higher CQI has been associated with a lower risk of cardiovascular disease (CVD),^7^ obesity,^4^ and hypertension,^8^ along with reductions in cardiometabolic risk factors.^9^

In recent years, research has highlighted the importance of carbohydrate intake in relation to the composition and function of the human gut microbiota.^10^ Gut microbiota has been increasingly recognized as an important factor in a myriad of health functions, including a range of physiologic processes that are fundamental to host well-being, such as energy homeostasis, glucose and fat metabolism, immunological activity, and neurobehavioral development.^11,12^

Despite the composition of the gut microbiota tending to be established by the age of 3–6 years and remaining highly stable through time, it may still be largely influenced by environmental and lifestyle factors throughout the lifespan, including in older age.^13^ In addition to age, health status has also been implicated in impacting the gut microbial composition of the host. Specifically, obesity or diabetes has been associated with lower diversity and richness of the gut microbiota.^14,15^ Hence understanding the influence of dietary intake, especially carbohydrate quality, on gut microbiota and cardiovascular risk in individuals with overweight or obesity in diverse populations remains of importance.

While the CQI brings together several dimensions of dietary carbohydrate quality and may be an effective tool for nutrition counseling, very few studies have examined its relationship with gut microbiota. Furthermore, evidence supports the impact of the gut microbiota as an environmental factor related to the progress of obesity and metabolic disturbances, even though the causal nature of this has not been completely understood.^16^

Given the current scarcity of literature describing the association between the quality of carbohydrate intake, gut microbiota, and clinical cardiometabolic variables, the aim of the present study was to cross-sectionally and longitudinally assess the associations between CQI, fecal microbiota, and cardiometabolic risk factors in a cohort of older adults with overweight/obesity and metabolic syndrome from a Mediterranean population.

## Results

### Characteristics of the study population

A total of 656 PREDIMED (PREvención con DIeta MEDiterránea)-Plus participants, with available 16S data at baseline and 1-year timepoints were included in the present study. After conducting quality filtering steps on 16S data, and after removing participants exposed to prior antibiotic treatment, the total number of matching participants was further reduced to 641. Finally, after excluding participants with total calorie intake outside the pre-specified energy limits, 617 participants were retained for the cross-sectional and longitudinal analyses.

The characteristics of the study population according to tertiles of baseline CQI are described in Table 1. Individuals distributed in the lowest tertile (T1) presented a CQI of 7.7 ± 1.3, whereas individuals distributed in highest tertile (T3) presented a CQI of 15.6 ± 1.3. The percentage of women was lower in T1 (37%) compared to T3 (56%). In addition, there were significant differences in the number of individuals distributed in the different recruiting centers across tertiles (*p* < 0.001). Individuals distributed in T1 presented with a higher waist circumference compared to those individuals distributed in T3 (109.1 ± 9.4 *versus* 106.1 ± 9.6 cm). In addition, low-density lipoprotein (LDL) cholesterol was lower in T1 (117.1 ± 31.3 mg/dL) compared to T3 (125.3 ± 32.1 mg/dL). Individuals distributed in T1 showed lower adherence to Mediterranean diet (MedDiet) compared to those individuals distributed in T2 and T3 (7.2 ± 2.1 *versus* 8.3 ± 2.5 and 9.3 ± 2.5, respectively). In addition, individuals distributed in T1 did less physical activity compared with those distributed in T3. Individuals distributed in T1 showed higher intake of carbohydrates (255.5 ± 61.6 g/day) compared to those distributed in T3 (238.6 ± 57.4 g/day), whereas regarding CQI components, individuals distributed in T1 consumed lower fiber compared to those distributed in T2 and T3 (22.6 ± 5.9 *versus* 26.0 ± 6.8 and 33.3 ± 8.3 g/day, respectively) and presented with higher glycemic index compared to those distributed in T2 and T3 (57.3 ± 3.5 *versus* 53.6 ± 4.7 and 52.3 ± 4.6, respectively). The ratio of whole grains to total grains was lower in individuals distributed in T1 (0.04 ± 0.1) compared to those distributed in T2 (0.2 ± 0.2) and T3 (0.5 ± 0.3) as well as the ratio of solid carbohydrate to solid + liquid carbohydrate.

**Table 1.** Characteristics of the study population according to tertiles (T) of baseline CQI.

Tertile (n)	T1 (222)	T2 (206)	T3 (189)	*p-*value
CQI	7.7 ± 1.3	11.4 ± 1.1	15.6 ± 1.3	
Women	83 (37.4)	96 (46.6)	106 (56.1)^a^	.001
Recruiting center				<.001
Alicante	26 (11.7)	60 (29.1)^a^	47 (24.9)^a^	
Barcelona	15 (6.8)	21 (10.2)	28 (14.8)^a^	
Reus	157 (70.7)	94 (45.6)^a^	89 (47.1)^a^	
Valencia	24 (10.8)	31 (15.0)	25 (13.2)	
Smoking status				.651
Former smoker	93 (41.9)	78 (37.9)	70 (37.0)	
Never smoked	99 (44.6)	100 (48.5)	98 (51.9)	
Current smoker	30 (13.5)	28 (13.6)	21 (11.1)	
Hypercholesterolemia prevalence	142 (64.0)	141 (68.4)	126 (66.7)	.613
Hypertension prevalence	189 (85.1)	168 (81.6)	143 (75.7)	.051
Diabetes prevalence	51 (23.0)	48 (23.3)	55 (29.1	.286
Age (years)	64.7 ± 5.2	65.2 ± 5.0	65.0 ± 4.3	.489
Body weight (kg)	88.5 ± 13.4	87.0 ± 12.6	85.9 ± 12.8	.132
Waist circumference (cm)	109.1 ± 9.4	108.0 ± 10.4	106.1 ± 9.6^a^	.009
BMI (kg/m^2^	32.9 ± 3.6	32.9 ± 3.5	32.8 ± 3.4	.955
Total cholesterol (mg/dL)	198.3 ± 36.8	200.4 ± 38.3	203.9 ± 37.8	.317
HDL-cholesterol (mg/dL)	48.5 ± 10.9	49.8 ± 10.8	50.4 ± 12.5	.226
LDL-cholesterol (mg/dL)	117.1 ± 31.3	121.1 ± 33.1	125.3 ± 32.1^a^	.048
Triglycerides (mg/dL)	172.9 ± 98.2	157.0 ± 90.3	153.6 ± 96.9	.860
Fasting plasma glucose (mg/dL)	116.6 ± 24.2	114.0 ± 24.0	115.1 ± 25.4	.547
Insulin (mU/mL)	20.5 ± 11.2	19.0 ± 11.0	19.2 ± 11.5	.377
HOMA-IR index	6.0 ± 3.8	5.5 ± 4.0	5.7 ± 4.4	.391
Glycated hemoglobin (%)	6.1 ± 0.8	6.0 ± 0.7	6.1 ± 0.8	.791
MedDiet adherence score	7.2 ± 2.1	8.3 ± 2.5^a^	9.3 ± 2.5^a,b^	<.001
Physical activity (METs min/day)	323.5 ± 301.9	365.1 ± 311.9	421.3 ± 396.0	.014
Total energy intake (kcal/day)	2500.4 ± 476.9	2447.4 ± 532.3	2477.3 ± 474.8	.543
Carbohydrate intake (g/day)	255.5 ± 61.6	240.0 ± 73.4^a^	238.6 ± 57.4^a^	.012
Fiber intake (g/day)	22.6 ± 5.9	26.0 ± 6.8^a^	33.3 ± 8.3^a,b^	<.001
Glycemic index	57.3 ± 3.5	53.6 ± 4.7^a^	52.3 ± 4.6^a,b^	<.001
Ratio whole total grain	0.04 ± 0.1	0.2 ± 0.2^a^	0.5 ± 0.3^a,b^	<.001
Ratio solid liquid carbohydrate	1.0 ± 0.04	1.0 ± 0.03^a^	1.0 ± 0.02^a,b^	<.001

CQI, carbohydrate quality index; BMI, body mass index; HDL, high-density lipoprotein; LDL, low-density lipoprotein; HOMA-IR, homeostasis model assessment of insulin resistance; MedDiet, Mediterranean diet; METs, metabolic equivalents of task. Data described as number (percentage) for categorical variables or mean ± standard deviation for numerical variables. Differences across tertiles are calculated using one-way ANOVA test or Pearson’s chi-squared test differences between tertiles are calculated using Student’s independent sample t-test or Pearson’s chi-squared test. ^a^ *p* < 0.05 vs T1, ^b^ *p* < 0.05 vs T2.

The characteristics of the study population according to tertiles of 1-year change in CQI are described in Table 2. Individuals distributed in T1 showed a reduction in CQI (−4.0 ± 2.0) whereas individuals distributed in T3 showed an increase in CQI (4.7 ± 2.1) after 1-year of lifestyle intervention. The percentage of individuals distributed in the intervention group was lower in T1 (30%) compared to T2 (48%) and T3 (75%). In addition, there were significant differences in the number of individuals distributed in the different recruiting centers across tertiles (*p* = 0.046). After 12 months of lifestyle intervention, individuals distributed in T1 showed a lower decrease in body weight compared to those individuals distributed in T2 and T3 (−1.3 ± 3.3 *versus* −2.4 ± 3.9 and − 4.6 ± 4.0 kg, respectively), a lower decrease in waist circumference compared to those distributed in T2 and T3 (−1.7 ± 4.9 *versus* −2.9 ± 4.9 and − 5.4 ± 6.0 cm, respectively), and a lower decrease in body mass index (BMI) compared to those distributed in T2 and T3 (−0.5 ± 1.2 *versus* −0.9 ± 1.5 and − 1.7 ± 1.5 kg/m^2^, respectively). A lower decrease in plasma levels of triglycerides after 1-year of lifestyle intervention was observed in the individuals distributed in T1 (−3.5 ± 76.6 mg/dL) compared to those distributed in T3 (−20.0 ± 88.6 mg/dL), as well as a lower decrease in the percentage of glycated hemoglobin (HbA1c) was observed in individuals belonging to T1 (−0.001 ± 0.6%) compared to T3 (−0.2 ± 0.4%). The increase in the adherence to MedDiet score after 1-year of lifestyle intervention was lower in those individuals distributed in T1 (2.2 ± 2.8) compared to those individuals distributed in T2 (3.9 ± 3.2) and T3 (5.8 ± 3.2). In addition, the increase in physical activity was lower in individuals distributed in T1 compared to those distributed in T2 and T3. Individuals distributed in T1 showed a lower decrease in carbohydrate intake after 1-year of follow up (−28.7 ± 68.5 g/day) compared to those individuals distributed in T3 (−45.0 ± 68.8 g/day), whereas regarding the change in individual CQI components following the 1-year lifestyle intervention, fiber intake decreased in individuals distributed in T1 (−2.7 ± 7.6 g/day) and increased in those distributed in T2 (3.1 ± 7.4 g/day) and T3 (10.2 ± 6.7 g/day), as well as the ratio of whole grains to total grains and the ratio of solid carbohydrate to solid + liquid carbohydrate. In addition, glycemic index increased (*p* < 0.001) in T1 (1.0 ± 4.4) and decreased in T2 (−1.3 ± 4.9) and T3 (−4.7 ± 4.8) after 1-year of lifestyle intervention.

**Table 2.** Characteristics of the study population according to tertiles (T) of CQI 1-year change.

Tertile (n)	T1 (234)	T2 (180)	T3 (203)	*p-*value
CQI change	−4.0 ± 2.0	−0.1 ± 0.8	4.7 ± 2.1	
Women	113 (48.3)	82 (45.6)	90 (44.3)	.696
Intervention group	70 (29.9)	87 (48.3)^a^	153 (75.4)^a,b^	<.001
Recruiting center				.046
Alicante	61 (26.1)	42 (23.3)	30 (14.8)^a^	
Barcelona	19 (8.1)	21 (11.7)	24 (11.8)	
Reus	118 (50.4)	98 (54.4)	124 (61.1)	
Valencia	36 (15.4)	19 (10.6)	25 (12.3)	
Smoking status				.079
Former smoker	80 (34.2)	79 (43.9)	83 (40.9)	
Never smoked	115 (49.1)	86 (47.8)	95 (46.8)	
Current smoker	39 (16.7)	15 (8.3)	25 (12.3)	
Hypercholesterolemia prevalence	158 (67.5)	114 (63.3)	138 (68.0)	.572
Hypertension prevalence	187 (79.9)	147 (81.7)	166 (81.8)	.856
Diabetes prevalence	61 (26.1)	47 (26.1)	46 (22.7)	.652
Body weight change (Kg)	−1.3 ± 3.3	−2.4 ± 3.9^a^	−4.6 ± 4.0^a,b^	<.001
Waist circumference change (cm)	−1.7 ± 4.9	−2.9 ± 4.9^a^	−5.4 ± 6.0^a,b^	<.001
BMI change (kg/m^2^	−0.5 ± 1.2	−0.9 ± 1.5^a^	−1.7 ± 1.5^a,b^	<.001
Total cholesterol change (mg/dL)	1.0 ± 48.3	2.8 ± 35.2	−2.7 ± 28.9	.369
HDL-cholesterol change (mg/dL)	1.3 ± 6.5	2.5 ± 7.9	2.7 ± 7.0	.070
LDL-cholesterol change (mg/dL)	−1.3 ± 27.3	0.3 ± 30.3	−2.4 ± 24.3	.661
Triglycerides change (mg/dL)	−3.5 ± 76.7	0.3 ± 73.8	−20.0 ± 88.6^a,b^	.028
Fasting plasma glucose change (mg/dL)	−3.0 ± 22.3	−3.2 ± 13.4	−5.5 ± 16.9	.335
Insulin change (mU/mL)	−2.5 ± 9.0	−1.5 ± 10.2	−3.4 ± 9.7	.191
HOMA−IR index change	−0.9 ± 3.8	−0.6 ± 3.3	−1.2 ± 3.2	.261
Glycated hemoglobin change (%)	0.001 ± 0.6	−0.03 ± 0.4	−0.2 ± 0.4^a,b^	.001
MedDiet adherence score change	2.2 ± 2.8	3.9 ± 3.2^a^	5.8 ± 3.2^a,b^	<.001
Physical activity change (METs min/day)	54.0 ± 323.7	122.2 ± 354.5^a^	172.5 ± 375.7^a^	.002
Total energy intake change (kcal/day)	−165.0 ± 476.3	−153.8 ± 454.6	−162.6 ± 480.1	.970
Carbohydrate intake change (g/day)	−28.7 ± 68.5	−34.2 ± 63.3	−45.0 ± 68.8^a^	.040
Fiber intake change (g/day)	−2.7 ± 7.6	3.1 ± 7.4^a^	10.2 ± 6.7^a,b^	<.001
Glycemic index change	1.0 ± 4.4	−1.3 ± 4.9^a^	−4.7 ± 4.8^a,b^	<.001
Ratio whole total grain change	−0.07 ± 0.3	0.2 ± 0.3^a^	0.6 ± 0.3^a,b^	<.001
Ratio solid liquid carbohydrate change	−0.003 ± 0.04	0.01 ± 0.03^a^	0.02 ± 0.03^a^	<.001

CQI, carbohydrate quality index; BMI, body mass index; HDL, high density lipoprotein; LDL, low density lipoprotein; HOMA-IR, homeostatic model assessment of insulin resistance; MedDiet, Mediterranean diet; METs, metabolic equivalents of task. Data described as number (percentage) for categorical variables or mean ± standard deviation for numerical variables. Differences across tertiles are calculated using one-way ANOVA test or Pearson’s chi-squared test, differences between tertiles are calculated using Student’s independent sample t-test or Pearson’s chi-squared test. ^a^ *p* < 0.05 vs T1, ^b^ *p* < 0.05 vs T2.

### Fecal microbiota alpha and beta diversity profiles associated with carbohydrate quality

**Figure 1.**
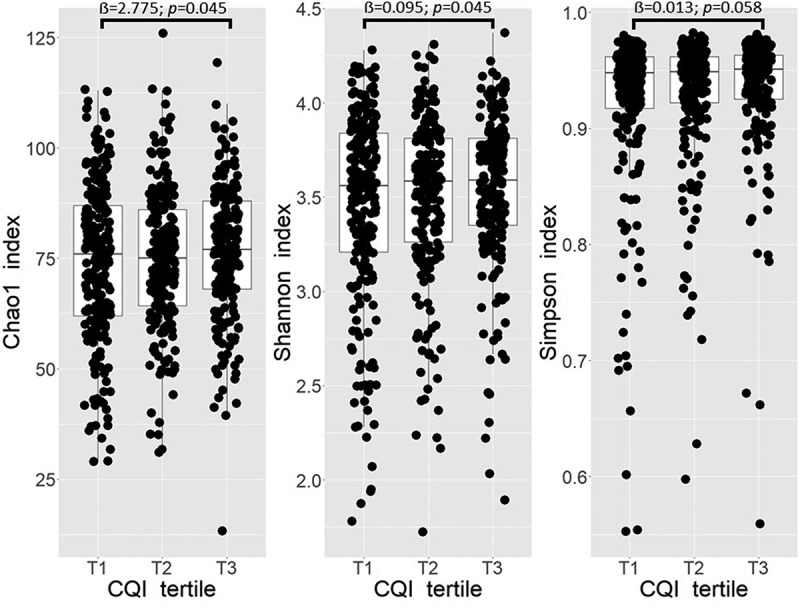
Boxplots representing the differences in alpha diversity indices Chao1, Shannon, and Simpson across tertiles (T) of baseline CQI. Linear regression was used to test the cross-sectional association between tertiles of baseline CQI and selected indices. Models were adjusted for recruiting center (Alicante, Barcelona, Reus, Valencia), smoking status (former smoker, never smoked, smoker), diabetes status, sex, age categories (below the median, < 65 years old; above the median, > 65 years old). T1 set as reference, *p* < 0.05 deemed as significant.

A significant positive association between Shannon index and T3 compared to T1 of baseline CQI was observed (*p* = 0.045). No significant association was observed between Chao1 index and T3 compared to T1 of baseline CQI, whereas Simpson index shown a *p*-value trend slightly close to significance (*p* = 0.058) (Figure 1). No significant associations between 1-year change in alpha diversity indices Chao1, Shannon and Simpson and tertiles of 1-year change in CQI were observed (Figure S1).

Principal components calculated over baseline centered log-ratio (clr) transformed taxonomic feature counts showed that PC1 and PC2 accounted for approximately 10.2% and 8.1% to the total variation, respectively. Principal component analysis (PCA) plot showed that the fecal microbiota samples did not cluster based on tertiles of baseline CQI (Figure S2). Principal components calculated over baseline and 1-year clr-transformed taxonomic feature counts shown that PC1 and PC2 account approximately 9.9% and 7.9% to the total variation, respectively. PCA plot showed that the fecal microbiota samples did not cluster based on tertiles of 1-year change in CQI (Figure S3).

The results of the permutational multivariate analysis of variance (PERMANOVA) test based on Aitchison distance did not show statistically significant differences across tertiles of baseline CQI (table S1), nor across tertiles of 1-year change in CQI (table S2).

### Taxonomic features and metabolic pathways associated with carbohydrate quality

Cross-sectionally, the multivariable associations between the highest tertile of baseline CQI (T3), compared to the lowest tertile (T1) as reference category, and baseline abundance of taxonomic features at genus level, showed a negative association between T3 and *Odoribacter* (FDR = 0.008), and T3 and uncultured *Rhodospirillales* genera (FDR = 0.036). A positive association was observed between T3 and *Faecalibacterium* (FDR = 0.097), and T3 and *Christensenellaceae* R7 group (FDR = 0.097) (Table 3). In addition, we observed a positive association between T3, and metabolic pathways related to peptidoglycan biosynthesis IV (PWY-6471) (FDR = 0.006), CMP-legionaminate biosynthesis I (PWY-6749) (FDR = 0.007), pyruvate fermentation to butanoate (CENTFERM-PWY) (FDR = 0.011), superpathway of *Clostridium acetobutylicum* acidogenic fermentation (PWY-6590) (FDR = 0.011), nitrate reduction VI (PWY490–3) (FDR = 0.034), β-(1,4)-mannan degradation (PWY-7456) (FDR = 0.041), glutaryl-CoA degradation (PWY-5177) (FDR = 0.042) (Table 4).

**Table 3.** Fecal microbiota genera associated with the highest tertile (T3) of baseline CQI.

Genera	ẞ Coef.	Std. err.	*p*-value	FDR
*Odoribacter*	−0.259	0.070	<.001	0.008
*Rhodospirillales* uncultured genus	−0.204	0.064	.001	0.036
*Faecalibacterium*	0.307	0.111	.006	0.097
*Christensenellaceae* R7 group	0.427	0.156	.006	0.097

Associations tested with generalized linear models. Recruiting center (Alicante, Barcelona, Reus, Valencia), smoking status (former smoker, never smoked, current smoker), diabetes status, sex, and age categories (below the median, < 65 years old; above the median, > 65 years old) set as fixed effect. Multiple testing correction performed with the Benjamini-Hochberg procedure and features with FDR < 0.1 reported (Reference: Tertile 1).

**Table 4.** Metabolic pathways associated with the highest tertile (T3) of baseline CQI.

MetaCyc ID	Pathway	ẞ Coef.	Std. err.	*p*-value	FDR
PWY-6471	Peptidoglycan biosynthesis IV	0.2325	0.0642	0.0003	0.0064
PWY-6749	CMP-legionaminate biosynthesis I	0.2955	0.0825	0.0004	0.0071
CENTFERM-PWY	Pyruvate fermentation to butanoate	0.4072	0.1200	0.0007	0.0109
PWY-6590	Superpathway of Clostridium acetobutylicum acidogenic fermentation	0.4172	0.1229	0.0007	0.0109
PWY490–3	Nitrate reduction_VI	0.2145	0.0716	0.0029	0.0338
PWY-7456	β-(1,4)-mannan degradation	0.2406	0.0823	0.0036	0.0409
PWY-5177	glutaryl-CoA degradation	0.3461	0.1187	0.0037	0.0415

Associations tested with generalized linear models. Recruiting center (Alicante, Barcelona, Reus, Valencia), smoking status (former smoker, never smoked, smoker), diabetes status, sex, age categories (below the median, < 65 years old; above the median, > 65 years old), and intervention group (CG, IG) set as fixed effect; participant ID set as random effect. Multiple testing correction performed with the Benjamini-Hochberg procedure and features with FDR < 0.1 reported (Reference: Tertile 1).

Longitudinally, the multivariable associations between the highest tertile (T3) of 1-year change in CQI, compared to the lowest tertile (T1) as the reference category, and 1-year change in the abundance of taxonomic features at genus level showed a negative association between T3 and *Monoglobus* (FDR = 0.065), and a positive association between T3 and *Butyrivibrio* (FDR = 0.065) (Table 5). No association was observed between metabolic pathways and T3.

**Table 5.** Fecal microbiota genera associated with the highest tertile (T3) of CQI 1-year change.

Genera	ẞ Coef.	Std. err.	*p*-value	FDR
*Butyrivibrio*	0.250	0.090	.006	0.065
*Monoglobus*	−0.188	0.069	.006	0.072

Associations tested with generalized linear models. Recruiting center (Alicante, Barcelona, Reus, Valencia), smoking status (former smoker, never smoked, smoker), diabetes status, sex, age categories (below the median, < 65 years old; above the median, > 65 years old), and intervention group (CG, IG) set as fixed effect; participant ID set as random effect. Multiple testing correction performed with the Benjamini-Hochberg procedure and features with FDR < 0.1 reported (Reference: Tertile 1).

### CQI-related genera associated with cardiovascular risk factors

**Figure 2.**
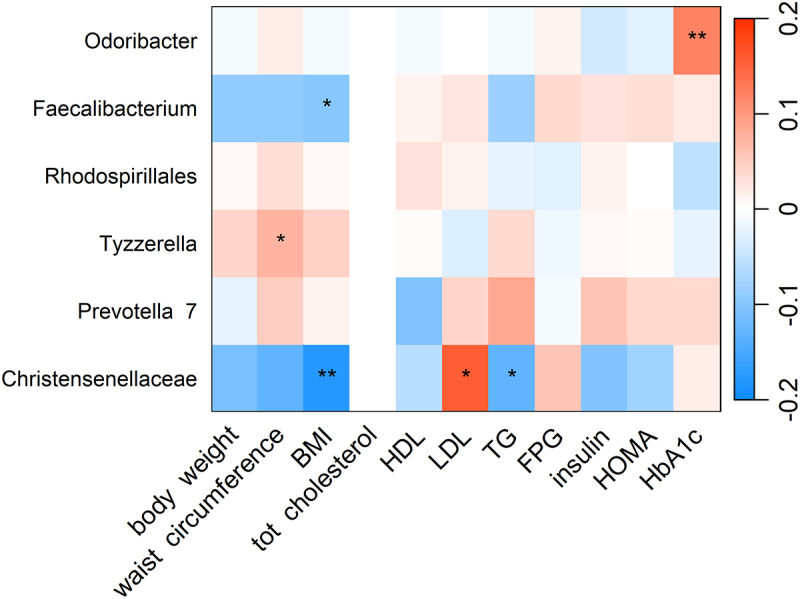
Heatmap showing the cross-sectional association between CQI-related genera and cardiovascular risk factors. Association tested by linear regression adjusted for recruiting center (Alicante, Barcelona, Reus, Valencia), smoking status (former smoker, never smoked, smoker), diabetes status, sex, age categories (below the median, < 65 years old; above the median, > 65 years old). For each cell, colors indicate the association coefficient with cardiovascular risk factors and asterisks denote significance. *p < 0.05; **p < 0.01. TG, triglycerides FPG, fasting plasma glucose; HDL, high-density lipoprotein; LDL, low-density lipoprotein; HOMA, homeostasis model assessment; HbA1c, glycated hemoglobin.

Among baseline CQI related genera, we observed a positive association between *Odoribacter* and glycated hemoglobin, whereas *Faecalibacterium* and *Christensenellaceae* R7 group were negatively associated with BMI. In addition, *Christensenellaceae* R7 group was negatively associated with triglycerides and positively associated with LDL cholesterol (Figure 2).

**Figure 3.**
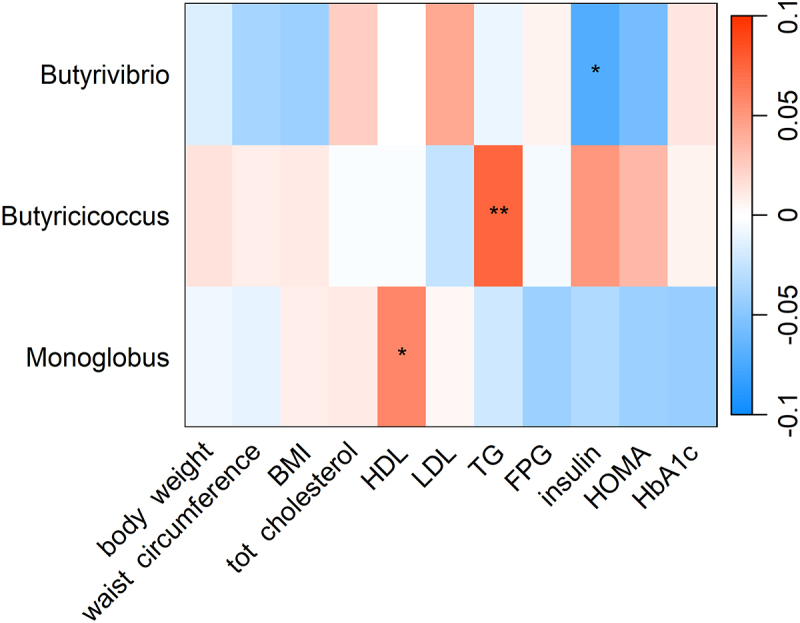
Heatmap showing the longitudinal association between 1-year change CQI-related genera and 1-year change in cardiovascular risk factors. Association tested with linear mixed model and adjusted for intervention group, timepoint, recruiting center (Alicante, Barcelona, Reus, Valencia), smoking status (former smoker, never smoked, smoker), diabetes status, sex, age categories (below the median, < 65 years old; above the median, > 65 years old). Participant ID was set as random effect. For each cell, colors indicate the association coefficient with cardiovascular risk factors and asterisks denote significance. *p < 0.05; **p < 0.01. TG, triglycerides FPG, fasting plasma glucose; HDL, high-density lipoprotein; LDL, low-density lipoprotein; HOMA, homeostasis model assessment; HbA1c, glycated hemoglobin.

Among CQI 1-year change related genera, we observed a negative association between 1-year change in *Butyrivibrio* abundance and 1-year change in insulin, and a positive association between 1-year change in *Monoglobus* abundance and 1-year change in high-density lipoprotein (HDL) cholesterol (Figure 3).

## Discussion

To our knowledge, this is the first study analyzing the relationship between CQI and fecal microbiota in Mediterranean population of elderly adults at high cardiovascular risk. Findings suggest that a higher quality of carbohydrate intake is associated with increased alpha diversity and abundance of specific genera, and part of this fecal microbial profile are related to better cardiometabolic health in older Spanish adults with overweight or obesity and metabolic syndrome.

The observed beneficial associations between CQI and cardiometabolic health align with previous evidence showing the consumption of carbohydrates based on individual quality traits, in particular higher intakes of total dietary fiber and whole grains, has a positive impact on population health outcomes, including mortality^5^. While the underlying mechanisms responsible for these observations remained incompletely understood, one possible explanation, as demonstrated by the present findings, may be modification of host gut microbiota. This connection is suggested in the present study, as higher CQI was related to better cardiometabolic health, based on associations with assessed clinical risk factors, as well as fecal microbiota. Specifically, in the present study, those individuals with higher CQI had lower waist circumference, and having a higher CQI was observed to be negatively associated with the abundance of members belonging to the Bacteroidetes and Proteobacteria phyla (i.e., *Odoribacter*, and *Rhodospirillales* uncultured genus) and positively associated with genera in the Firmicutes phylum (i.e., *Faecalibacterium* and *Christensenellaceae* R7 group). Where, lower abundance of *Odoribacter* was associated with lower glycated hemoglobin, and higher abundance of *Faecalibacterium* and *Christensenellaceae* R7 group were related to lower BMI levels. In addition, we observed that higher baseline CQI was positively associated with some pathways related with the metabolism of butyrate precursors (CENTFERM-PWI, PWI-6590) and plants-origin molecules (PWI490–3, PWI-7456).

Bacteroidetes (Gram-negative) and Firmicutes (Gram-positive) are generally the dominant phyla within the gut, with other phyla often comprising 10% or less of the gut microbiota, and largely occupy different functional niches in the gut ecosystem.^17^ As a result, differences between individuals in their relative proportion can lead to large differences in function, with relevance for host health.^18^ Members of the Bacteroidetes phylum have been associated with metabolic diseases.^18^ While there is substantial diversity among members of the Bacteroidetes phylum, they share certain attributes, for instance their superlative ability to utilize polysaccharides, and compared to bacteria of other phyla, Bacteroidetes members encode a proportionally high number of carbohydrate-active enzymes that enable use of both dietary and host mucosal glycans.^18^ Interestingly, the Bacteroidetes genus *Odoribacter* has also been linked to metabolic health benefits based on previous studies on metabolism-related pathologies, such as obesity, metabolic syndrome, and diabetes.^19–23^ However, the role of *Odoribacter* abundance in metabolic disorders has been inconsistent. Notably, *Odoribacter* has previously been shown in an older diabetes-free Spanish population to be negatively associated with homeostasis model assessment (HOMA) of insulin resistance.^24^ Yet, the present findings do not corroborate this as higher carbohydrate quality intakes were associated with lower amounts of *Odoribacter*, furthermore reduced abundance of *Odoribacter* was related to elevated glycated hemoglobin. However, it is unclear whether these associations are successive or possibly due to the nature of the study population having overweight or obesity and metabolic syndrome as it is known that these disease states alter the microbiota profile of the gut. As such, reverse causation may play a role in these observations as individuals with higher glycated hemoglobin levels, greater risk of type 2 diabetes, and/or overweight or obesity may initiate a dietary pattern with higher carbohydrate quality.

Recently, in the Firmicutes phylum, the family *Christensenellaceae* has emerged as an important player in human health where the abundance in the human gut has been inversely related to host BMI in different populations.^25,26^ While evidence is limited, a positive relationship has previously been observed between adherence to a MedDiet characterized by a high consumption of fiber-rich foods, and *Christensenellaceae*.^27^ Similarly, the present study found CQI to be associated with higher levels of *Christensenellaceae* R7 group and substantiates previous findings with leanness, as indicated by the inverse relationship observed with BMI.

Mechanistically, complex carbohydrates have been shown to shape the composition of the gut microbiome by providing a nutrient source for particular microbes, resulting in their degradation and fermentation in the large intestine.^28^ This fermentation of complex carbohydrates can also impact gut health through produced metabolites.^28^ Among these bacteria-derived metabolites, short chain fatty acids (SCFAs), namely acetate, propionate, and especially butyrate, have been linked to a broad range of health promoting activities, including energy homeostasis, hypocholesterolemia, anti-inflammatory, anti-obesity, anti-angiogenesis, and antioxidant.^29,30^ A MedDiet, characterized by a high consumption of fiber-rich food, has previously been correlated with higher levels of SCFAs and SCFA-producing bacteria.^27^ Similarly, the present study we observed relationships between CQI and the butyrate-producing microbiota genera *Faecalibacterium*, and *Butyrivibrio*. The connection between higher CQI and the abundance of *Faecalibacterium* coupled with the lower BMI aligns with previous findings.^31^ Accordingly, we also observed that higher carbohydrate quality was positively associated with metabolic pathways related with SCFA production and molecules from plant-origin, highlighting the potential role that MedDiet, and higher adherence to this dietary pattern, has on improving carbohydrate quality intake and consequent potential beneficial cardiometabolic outcomes mediated by the gut microbiota. Over the 1-year period, increasing CQI by almost 5 points compared to a decrease of 4 points was positively associated with *Butyrivibrio* genus abundance. Examination of the diversity within the Firmicutes reveals a vast diversity in genomes and capacities, which may partly explain differing responses even in equivalent or comparable environmental conditions in their hosts. Nonetheless, the present observed changes in CQI were related to improvements in cardiometabolic risk factors, specifically greater weight loss and decreased waist circumference, BMI, and glycated hemoglobin, which align with common findings seen with butyrate-production and presence.^30,32^

Limitations of the present study should be acknowledged. First, given the observational nature of the study design causality cannot be ascertained. Second, given the study participants are older men and women with overweight/obesity and metabolic syndrome from a Mediterranean country, they are not necessarily representative of the general population. However, populations at high risk of cardiometabolic diseases represent an important proportion of the globe and hence findings of the present study may be generalizable to those community-dwelling older adults who may highly benefit from approaches to support good health. Furthermore, the homogeneity among participants reduces the likelihood of misclassification bias, reduces potential confounding, and increases internal validity. Third, CQI was determined based on self-reported data collected from a semiquantitative food frequency questionnaire (FFQ), which may be subject to some degree of measurement error. However, use of a FFQ is considered an appropriate approach to assess food and nutrient intake in large cohorts and moreover, the FFQ used in this adult cohort, has been repeatedly validated and widely used.^33,34^ Fourth, methodologically, the use of 16S sequencing provides relative rather than absolute quantitative results and generally limits the taxonomic profiling to genus-level resolution as the primers used for amplification bind to regions that may not be completely conserved across all bacteria, hence it typically does not allow for differentiation between closely related bacteria at the species level.^35^ This lack of data on species-level taxonomy does not allow for further inference of the pathways associated with relationships observed. Nonetheless, 16S sequencing does allow for the feasibility of analyzing a large number of samples and databases are established and well-curated, whereas those for more advanced and expensive techniques (i.e., shotgun metagenomic sequencing) are relatively new and still developing.^36,37^ Further, it has been shown that 16S rRNA data can yield accurate disease prediction results in comparison to shotgun data.^37^ Lastly, as in any observational study, some residual confounding might not have been completely excluded. Yet, analyses were adjusted for major potential confounders. For this reason, residual confounding is not considered to be a likely important cause of the observed findings.

Despite these limitations, this study has several strengths. One key strength of this study is that, to the authors’ knowledge, this is the first study to examine the relationship between quality of carbohydrate consumption (CQI) and gut microbiota in subjects at high CVD risk, which may be used as a foundation for future studies and informing practice in this field. Utilization of a multidimensional CQI that has the possibility of dynamically capturing changes in risk factors provides a more comprehensive approach compared to a unidimensional assessment of carbohydrate quality. Additionally, the relatively large cohort sample size, the prospective analysis with a year-long follow-up period, use of validated questionnaires and methods, and the use of multivariable analyses, adjusted for relevant confounders to minimize the possibility of reverse causality bias add to the robustness of the present findings.

## Patients and methods

### Study design and population

This study integrates a cross-sectional and longitudinal analysis of baseline and 1-year timepoint data from a cohort of participants within the framework of the PREDIMED-Plus lifestyle intervention study. This trial aims to assess the long-term effects of an intensive weight loss lifestyle intervention based on an energy-restricted MedDiet, physical activity promotion, and behavioral support (intervention group), *versus* a control group following an *ad libitum* MedDiet without any advice to increase physical activity.^38^ The trial was registered at the International Standard Randomized Controlled Trial (Number: ISRCTN89898870 -date of registration: 2014) and approved by the research ethics committees of all participating institutions. A detailed protocol is available on the website http://www.predimedplus.com/. Eligible participants were men and women (aged 55–75 years), without documented history of CVD at enrollment, with overweight/obesity (BMI ≥25 and <40 kg/m^2^) and who met at baseline at least three components of the metabolic syndrome.

The present study encompasses a subsample of participants from the PREDIMED-Plus recruiting centers of Reus, Barcelona (Institut Hospital del Mar d’Investigacions Mèdiques, IMIM), Alicante, and Valencia, not exposed to antibiotic treatment prior to stool sample collection and with available fecal microbiota 16S data at both timepoints after quality filtering steps. Participants who did not complete the FFQ at baseline and after 1-year of follow-up or with total calorie intake outside the pre-specified energy limits (women <500 and > 3,500 kcal/day, and men < 800 and > 4,000 kcal/day) were excluded from the analyses.^39^

### General assessments, anthropometric and blood biochemical measurements

A general questionnaire was administered to collect data on socio-demographics and medical conditions. Leisure time physical activity was measured by the validated Regicor Short Physical Activity Questionnaire.^40^ Waist circumference was measured midway between the lowest rib and the iliac crest using an anthropometric tape, body weight was measured twice using high-quality electronic calibrated scales and height was measured twice using a wall-mounted stadiometer. Blood pressure was measured in triplicate using a validated semiautomatic oscillometer (Omron HEM-705CP, Kyoto, Japan). The mean of anthropometric and blood pressure measurements was used for the analyses.

Blood samples were collected after an overnight fast, aliquoted, and stored at − 80°C until further analyses. Circulating levels of glucose, total cholesterol, HDL cholesterol and triglycerides were measured using standard enzymatic methods. LDL cholesterol was calculated with the Friedewald formula (whenever triglycerides were <300 mg/dL). Insulin was centrally measured by an electrochemiluminescence immunoassay using an Elecsys immunoanalyzer (Roche Diagnostics, Meylan, France). Insulin resistance was estimated at baseline using the HOMA index.^41^

### Dietary assessment and carbohydrate quality index

Dietary intake was assessed using the Spanish version of the validated 143-item semiquantitative FFQ,^34^ administered by trained dietitians during face-to-face visits at baseline and at 1-year follow-up. Participants reported their average frequency and quantity of foods consumed during the previous year. The intake of each item was calculated by multiplying a typical portion size by frequency of consumption (9 possible responses ranging from never to > 6 times/day). Spanish food composition tables^42^ were used to derive nutrient (sodium, saturated and trans fatty acids), fiber, alcohol (g/day), and total energy intake (kcal/day), as well as determine consumption of specific food groups, such as fruits and vegetables (g/day). Glycemic index and load were determined using International tables.^43^ Overall adherence to the MedDiet was assessed using a validated 17-point scale.^44^

CQI was calculated from dietary intake information obtained from the semiquantitative FFQs.^9^ Specifically, this index was constructed based on 4 items: (i) total dietary fiber intake (g/d); (ii) glycemic index 24; (iii) whole-grain/total grain ratio, and (iv) solid carbohydrate/total carbohydrate ratio. Total grains were estimated including whole grains, refined grains, and their derived products. Liquid carbohydrate intake was estimated as sugar-sweetened beverages and fruit juice consumption, while solid carbohydrate intake included the rest of the carbohydrates contained in solid foods.

To determine the CQI, participants were stratified into quintiles for each of the CQI components, and values ranging from 1 point for the first quintile to 5 points for the fifth quintile were assigned, except for the glycemic index component, which was inversely weighted (1 point for the fifth quintile and 5 points for the first quintile). All values were then added to calculate the CQI (score index ranging from 4 to 20), with higher values representing better carbohydrate quality.^9^ CQI was used to categorize the study population cross-sectionally, according to tertiles of baseline CQI, and longitudinally according to tertiles of 1-year change (deltas) in CQI.

### Stool samples collection, DNA extraction and 16S amplicon sequencing

Steps regarding stool samples collection, storage, and processing; microbial DNA extraction from feces; amplicon libraries preparation; 16S sequencing procedure; pipeline utilized to obtain the final data; are detailed described elsewhere.^45^

### Statistical analyses

16S data was analyzed using R (version 4.2.1) and R Studio (version 2022.07.1). A cut off value of 10% of prevalence at the genus level on the absolute abundances of the Amplicon sequence variants (ASV) counts was used to remove ASV with a prevalence ≤ 10% between samples. In order to probe the drivers of fine-scale bacterial population dynamics, we tested the possible effects of potential covariates on the fecal bacterial community composition adjusting further statistical models for recruiting center (Alicante, Barcelona, Reus, Valencia), smoking status (former smoker, never smoked, current smoker), diabetes status, sex, age categories (below the median, <65 years old; above the median, >65 years old). For longitudinal assessments we also adjusted for intervention group, and timepoint.

Fecal microbiota alpha diversity was assessed on absolute abundance raw counts by calculating Chao1, Shannon, and Simpson indices.^46–48^ Linear regression was performed to test cross-sectionally the association between calculated alpha diversity indices and tertiles of baseline CQI, and to test longitudinally the association between 1-year change in calculated alpha diversity indices and tertiles of 1-year change in CQI. A clr-transformation over taxonomic feature counts at genus level was performed to deal with the compositionality of 16S sequencing data^49^ prior conducting PCA and evaluate cross-sectionally the fecal microbiota distribution of the study population according to tertiles of baseline CQI, and longitudinally to evaluate the fecal microbiota distribution of the study population according to tertiles of 1-year change in CQI.

Beta diversity was calculated in terms of Euclidean distance over clr-transformed counts (Aitchison distance)^50^ at genus level and PERMANOVA performed using “adonis2” function of vegan package to test cross-sectionally differences in microbiota dissimilarity across tertiles of baseline CQI, and to test longitudinally differences in microbiota dissimilarity across tertiles of 1-year change in CQI setting participant ID as groups within which to constrain permutations.

Multivariable association between tertiles and microbial metataxonomic features was assessed cross-sectionally and longitudinally using the R package MaAsLin2^51^ (version 1.10.0). General linear models were performed on clr-transformed counts at the genus level without setting any additional normalization or transformation. Both in the cross-sectional and longitudinal analysis the lowest tertile (T1) of baseline CQI or 1-year change in CQI, respectively, was selected as the reference in order to explore those features associated with the highest tertile (T3). Multiple testing correction was performed using Benjamini-Hochberg procedure, and results with FDR < 0.1 were reported. For the longitudinal setup, participant ID was specified as a random effect parameter to address the non-independence between samples belonging to the same participant.

Multiple linear regression and multiple linear mixed models were performed to test the association between significant genera and CVD risk factors.

The baseline clinical characteristics of the study population and changes were described according to tertiles of baseline and 1-year change in CQI, respectively. Numerical variables were considered normally distributed according to the central limit theorem and described as means and standard deviations, whereas categorical variables were described as numbers and percentages. Differences across tertiles were tested with Pearson’s chi square test or one-way analysis of variance (ANOVA) as appropriate, whereas differences between tertiles were tested with independent-sample student’s t-test or Pearson’s chi square test. A *p-*value <0.05 was deemed as significant.

Phylogenetic Investigation of Communities by Reconstruction of Unobserved States package (PICRUSt2)^52^ was used to perform an inferential analysis of the functional potential of the microbiome. Demultiplexed sequences were parsed and used as input for PICRUSt2 to generate a table of inferred per-sample MetaCyc pathways^53^. Counts were clr-transformed, and cross-sectional and longitudinal association with tertiles assessed using MaAsLin2 and same settings used for taxonomic features counts.

## Conclusions

In conclusion, in this observational study, consumption of higher quality carbohydrates was observed to be associated with greater alpha diversity, and abundance of genera related to beneficial cardiometabolic outcomes. These findings provide valuable information in possible mechanistic pathways implicated in the mediation that gut microbiota have in the associations observed between the quality of carbohydrates and health, and to support the use of carbohydrate dietary recommendations with a microbiota-oriented therapeutic strategy to modulate cardiometabolic disease risk and improve health.

Future studies with a comprehensive set of metabolomics, metagenomics, transcriptomics, and longer follow-up with intermediate time points and investigation of fecal microbiota as a possible mediating factor in the diet-health relationship pathway, would provide a better understanding of the association between CQI and fecal microbiota and the subsequent impact on health. Furthermore, randomized controlled trials are warranted as they would aid in establishing causality.

Largely, findings from the present study contribute to understanding the impact of the quality of dietary carbohydrate on the human fecal microbiota, which may ultimately help understand relationships between carbohydrate quality, microbial populations, and health with potential to better inform future public health strategies.

## Abbreviations

ANOVA: one-way analysis of variance
ASV: amplicon sequence variant
BMI: body mass index
clr: centered log-ratio
CQI: carbohydrate quality index
CVD: cardiovascular disease
FFQ: food frequency questionnaire
HDL: high-density lipoprotein
HOMA: homeostasis model assessment
LDL: low-density lipoprotein
MedDiet: Mediterranean diet
PCA: principal component analysis
PERMANOVA: permutational multivariate analysis of variance
PREDIMED: PREvención con DIeta MEDiterránea
SCFA: short chain fatty acids
T: tertile

## Supplementary Material

Supplemental Material

## Data sharing

The datasets generated and analyzed during the current study are not publicly available due to data regulations and for ethical reasons, considering that this information might compromise research participants’ consent because our participants only gave their consent for the use of their data by the original team of investigators. However, collaboration for data analyses can be requested by sending a letter to the PREDIMED-Plus steering Committee (predimed_plus_scommittee@googlegroups.com). The request will then be passed to all the members of the PREDIMED-Plus Steering Committee for deliberation.
