# Supplementary material for: Carbohydrate quality, fecal microbiota and cardiometabolic health in older adults: a cohort study
Source: Gut Microbes. 2023 Aug 23;15(2):2246185. doi: 10.1080/19490976.2023.2246185 (PMC10449004; doi:10.1080/19490976.2023.2246185)
Supplement: Supplemental Material [file KGMI_A_2246185_SM4550.docx]

**Supplementary File**

**Supplementary figure 1**. Boxplots representing the difference in 1-year change in alpha diversity indices Chao1, Shannon, and Simpson across tertiles (T) of 1-year change in CQI.

**Supplementary figure 2**. Plot of principal components calculated over baseline CLR-transformed taxonomic feature counts according to tertiles (T) of baseline CQI

**Supplementary figure 3**. Plot of principal components calculated over baseline and 1-year CLR-transformed taxonomic feature counts according to tertiles (T) of 1-year change in CQI

**Supplementary table 1**. Results of PERMANOVA test based on Aitchison distance over baseline taxonomic feature counts and population stratified according to tertiles of baseline CQI

**Supplementary table 2**. Results of PERMANOVA test based on Aitchison distance over baseline and 1-year taxonomic feature counts and population stratified according to tertiles of 1-year change in CQI

**Supplementary figure 1.** Boxplots representing the difference in 1-year change in alpha diversity indices Chao1, Shannon, and Simpson across tertiles (T) of 1-year change in CQI. Linear regression used to test the longitudinal association between tertiles and indices. Model adjusted for intervention group (CG, IG), recruiting center (Alicante, Barcelona, Reus, Valencia), smoking status (former smoker, never smoker, smoker), diabetes status, sex, age categories (below the median, < 65 years old; above the median, > 65 years old). T1 set as reference; *p* < 0.05 deemed ad significant.


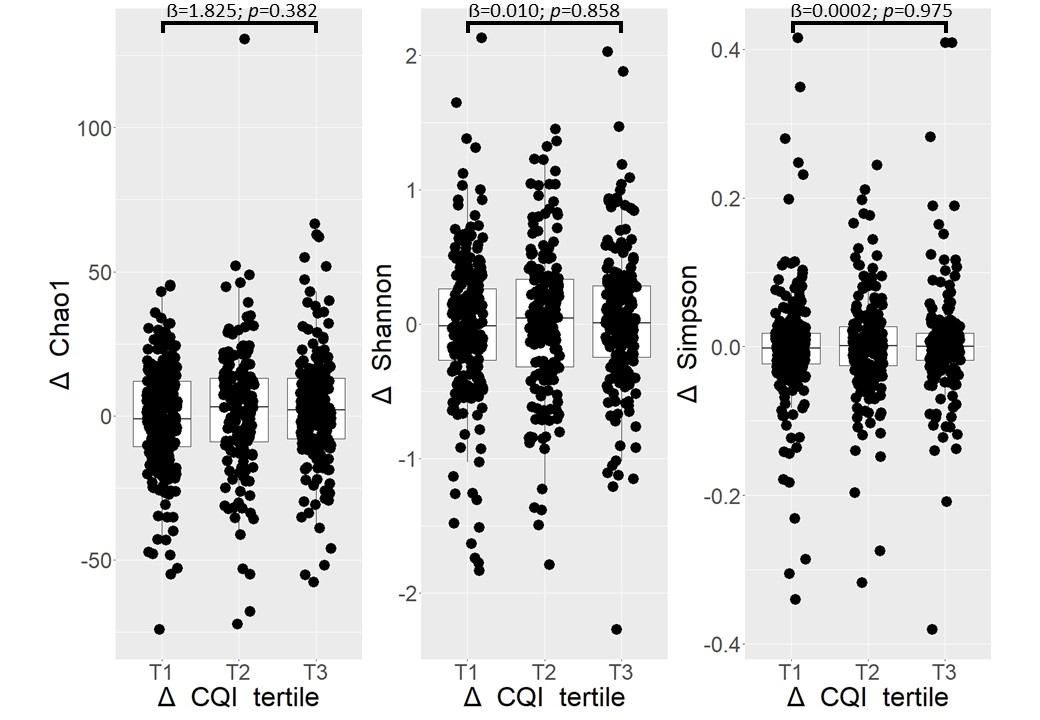


**Supplementary figure 2.** Plot of principal components calculated over baseline CLR-transformed taxonomic feature counts according to tertiles (T) of baseline CQI


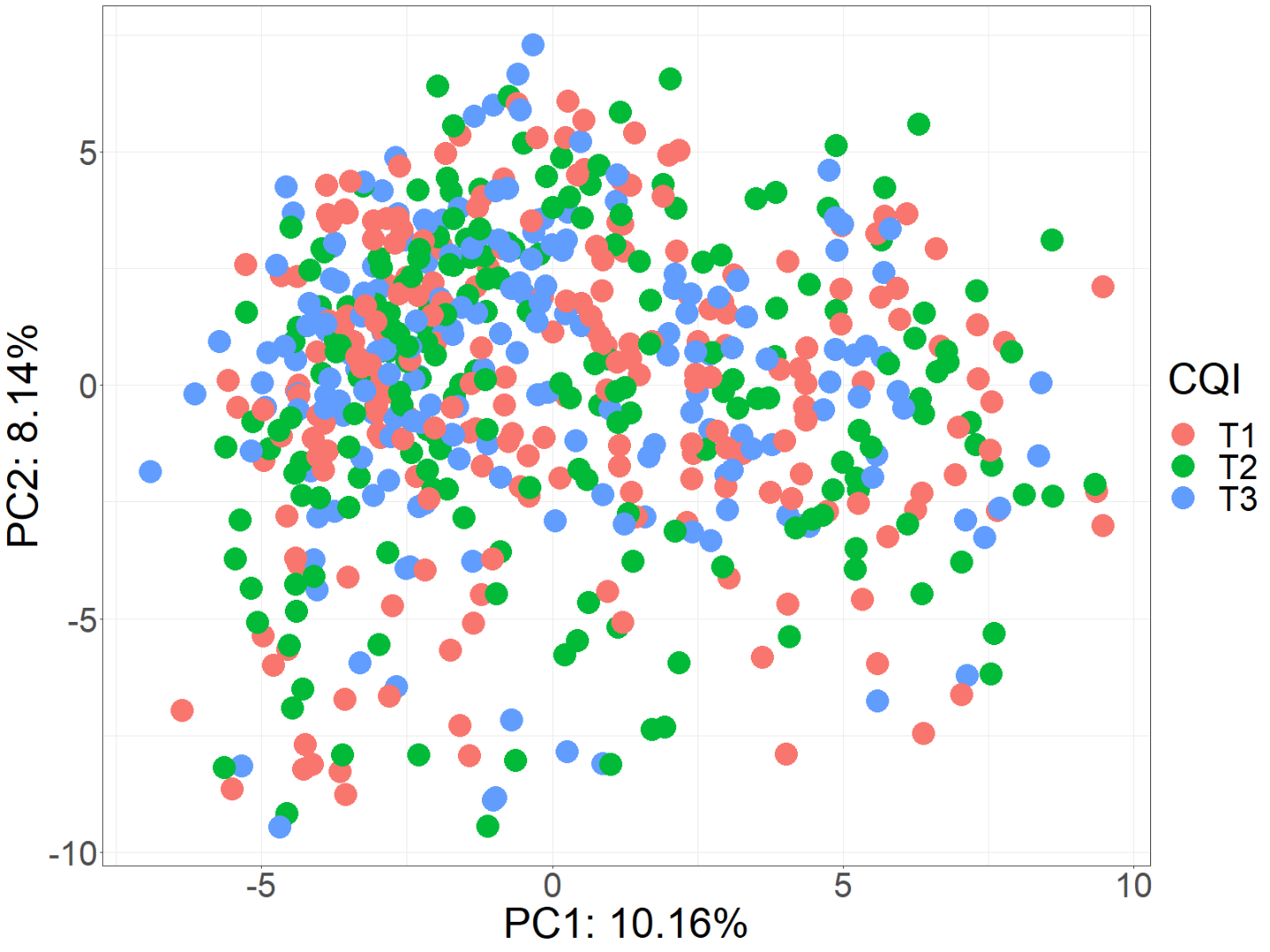


**Supplementary figure 3.** Plot of principal components calculated over baseline and 1-year CLR-transformed taxonomic feature counts according to tertiles (T) of 1-year change in CQI


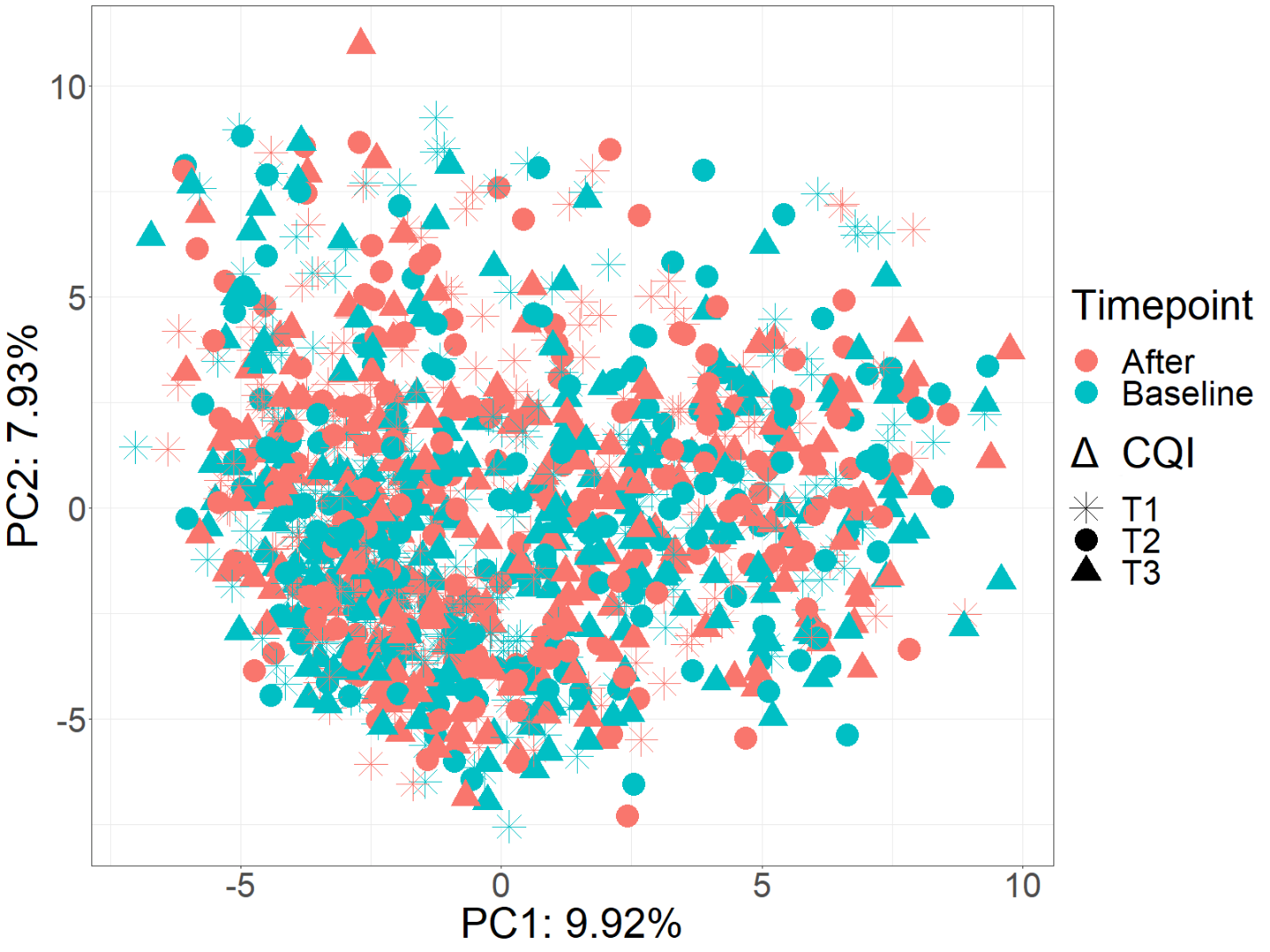


**Supplementary table 1.** Results of PERMANOVA test based on Aitchison distance over baseline taxonomic feature counts and population stratified according to tertiles of baseline CQI. Model adjusted for recruiting center (Alicante, Barcelona, Reus, Valencia), smoking status (former smoker, never smoker, smoker), diabetes status, sex, age categories (below the median, <6 5 years old; above the median, > 65 years old). Pr(>F) < 0.05 deemed as significant.

|  | **Df** | **Sum of sqs** | **R2** | **F** | **Pr(>F)** |
| --- | --- | --- | --- | --- | --- |
| CQI tertile | 2 | 320 | 0.00400 | 1.2732 | 0.068 |
| Recruiting center | 3 | 2694 | 0.03368 | 7.1541 | 0.001 |
| Smoke status | 2 | 340 | 0.00425 | 1.3552 | 0.050 |
| Sex | 1 | 233 | 0.00291 | 1.8548 | 0.004 |
| Age category | 1 | 172 | 0.00215 | 1.3730 | 0.085 |
| Diabetes | 1 | 157 | 0.00197 | 1.2525 | 0.119 |
| Residual | 606 | 76059 | 0.95104 |  |  |
| Total | 616 | 79975 | 1.00000 |  |  |

**Supplementary table 2.** Results of PERMANOVA test based on Aitchison distance over baseline and 1-year taxonomic feature counts and population stratified according to tertiles of 1-year change in CQI. Model adjusted for intervention group (CG, IG), timepoint, recruiting center (Alicante, Barcelona, Reus, Valencia), smoking status (former smoker, never smoker, smoker), diabetes status, sex, age categories (below the median, < 65 years old; above the median, > 65 years old). Participant ID set as random effect. Pr(>F) < 0.05 deemed as significant.

|  | **Df** | **Sum of sqs** | **R2** | **F** | **Pr(>F)** |
| --- | --- | --- | --- | --- | --- |
| CQI change tertile | 2 | 486 | 0.00302 | 1.9194 | 0.002 |
| Timepoint | 1 | 171 | 0.00106 | 1.3478 | 0.001 |
| Recruiting center | 3 | 4414 | 0.02738 | 11.6125 | 0.002 |
| Smoke status | 2 | 522 | 0.00324 | 2.0598 | 0.002 |
| Sex | 1 | 403 | 0.00250 | 3.1783 | 0.002 |
| Age category | 1 | 237 | 0.00147 | 1.8738 | 0.002 |
| Diabetes | 1 | 232 | 0.00144 | 1.8308 | 0.002 |
| Intervention group | 1 | 182 | 0.00113 | 1.4339 | 0.002 |
| CQI change tertile*Timepoint | 2 | 111 | 0.00069 | 0.4388 | 0.853 |
| Residual | 1219 | 154440 | 0.95808 |  |  |
| Total | 1233 | 161197 | 1.00000 |  |  |
